# Supplementary figures and images for: Quantifying risk factors associated with light-induced potato tuber greening in retail stores
Source: PLoS One. 2020 Sep 18;15(9):e0235522. doi: 10.1371/journal.pone.0235522 (PMC7500657; doi:10.1371/journal.pone.0235522)

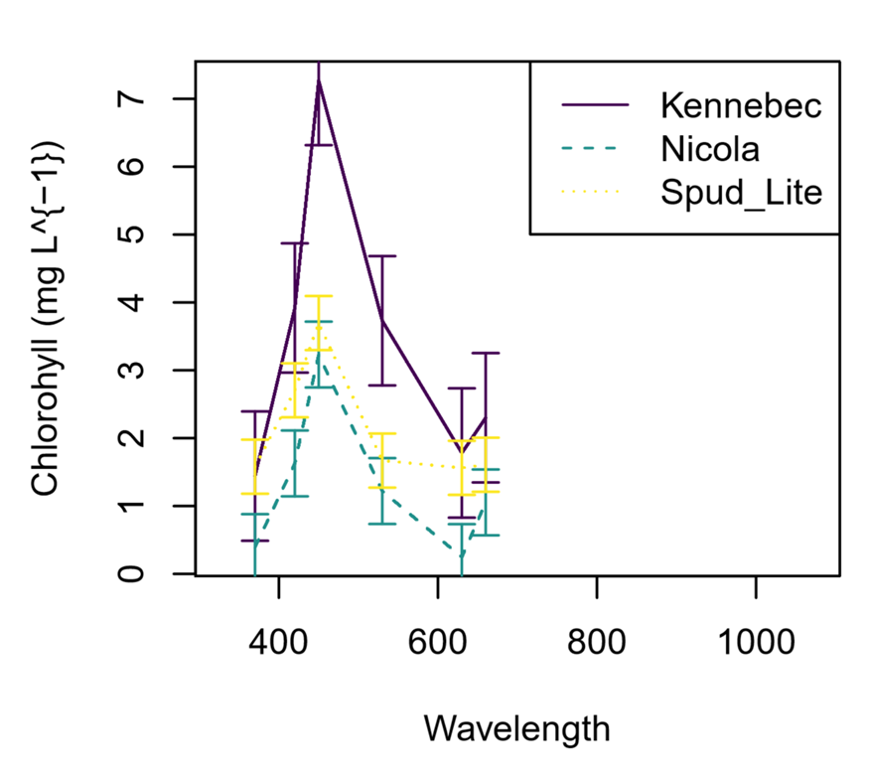

Supplement: S1 Fig — The weighting factor was calculated using the chlorophyll concentration of Nicola, after 120 hours of light exposure, at different light wavelengths (370, 420, 450, 530, 620, 660, and 735 nm). Given the observed chlorophyll, values were linearly interpolated for all wavelengths (325–735 nm) between each pair of points. (TIF) [file pone.0235522.s001.tif]

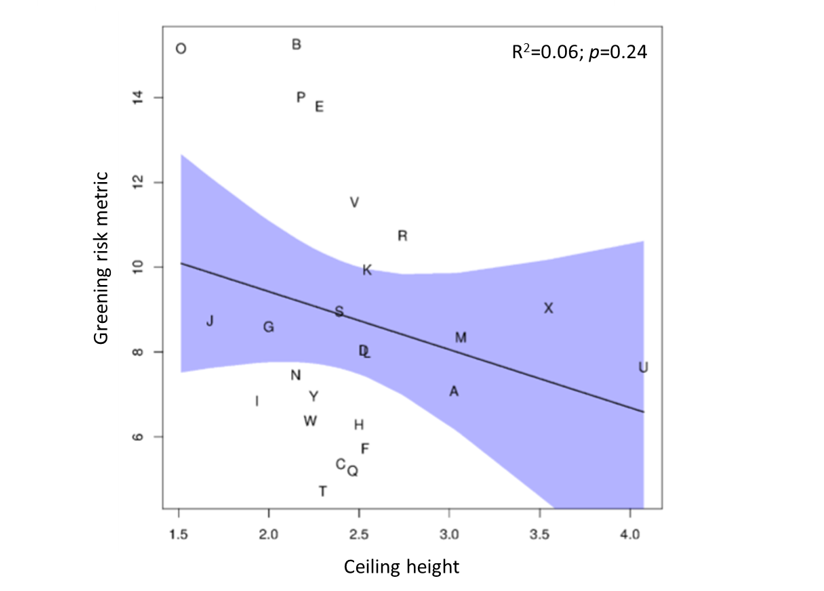

Supplement: S2 Fig — The shaded areas are the 95% confidence and prediction intervals. Each letter represents an individual retail store. (TIF) [file pone.0235522.s002.tif]

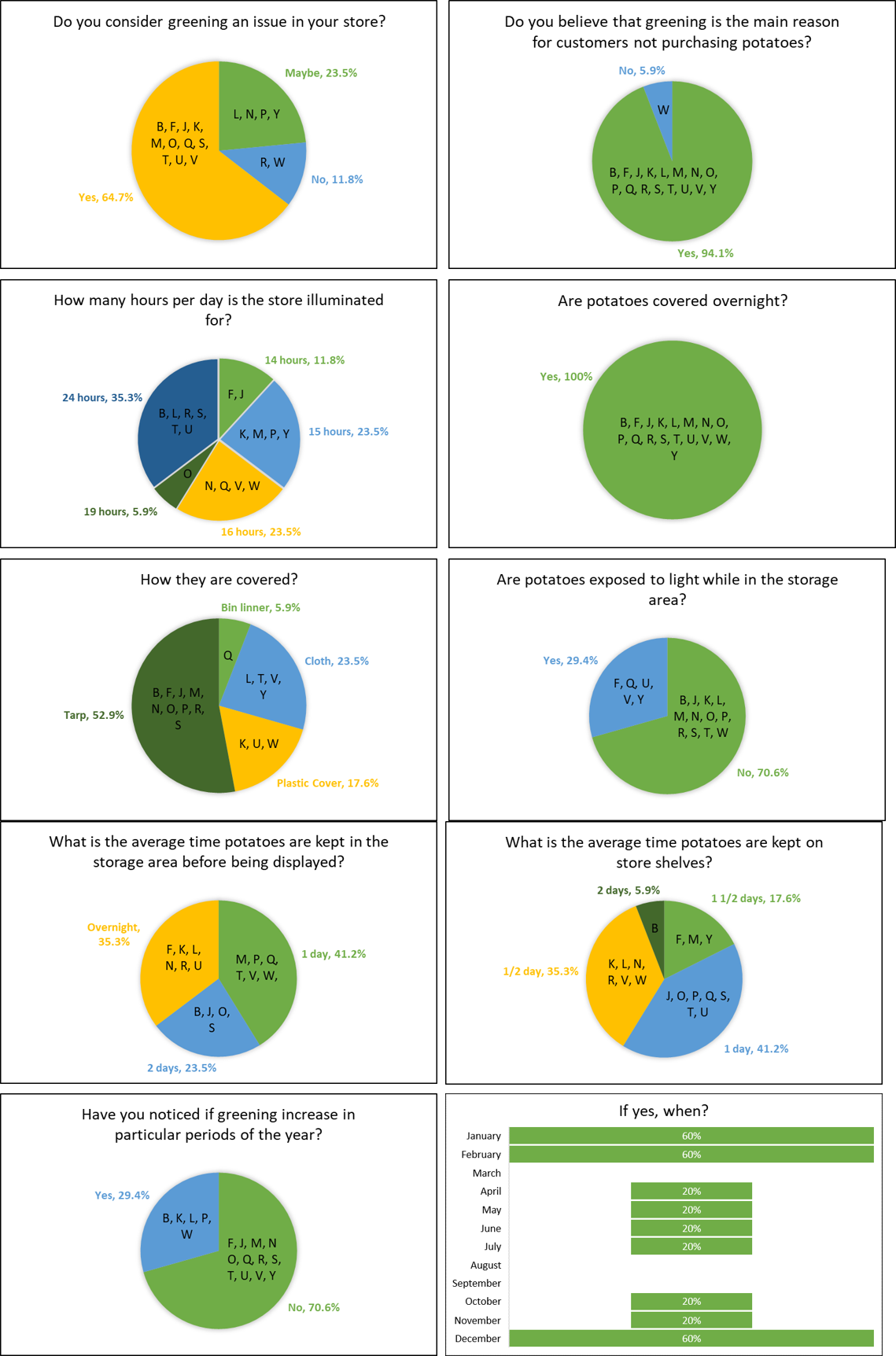

Supplement: S3 Fig — Each letter represents an individual retail store. (TIF) [file pone.0235522.s003.tif]

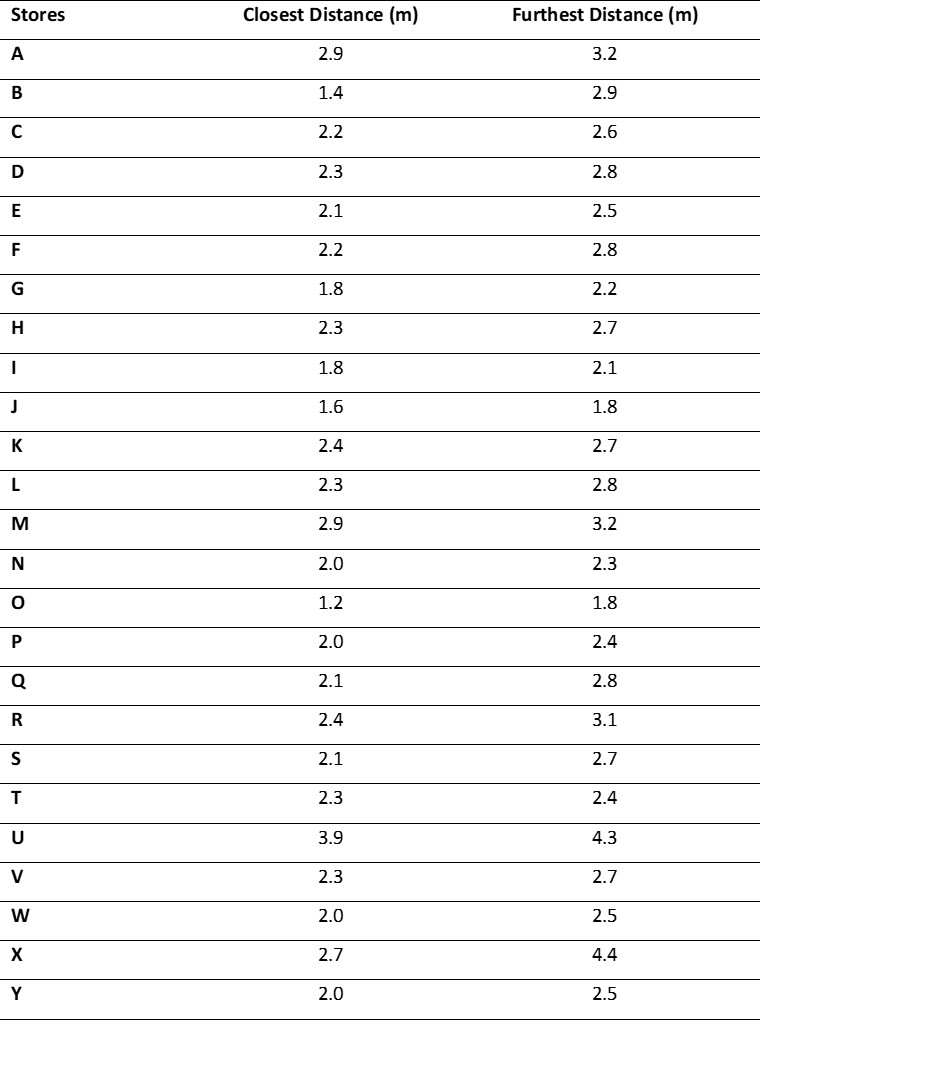

Supplement: S1 Table — Data represent the closest and furthest distance measured. Values are presented in meters (m). (TIF) [file pone.0235522.s004.tif]

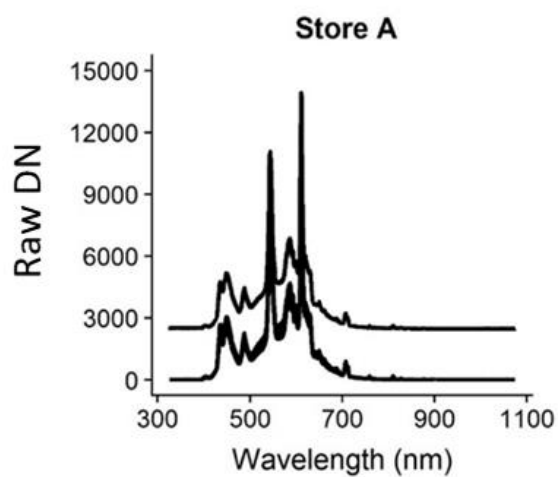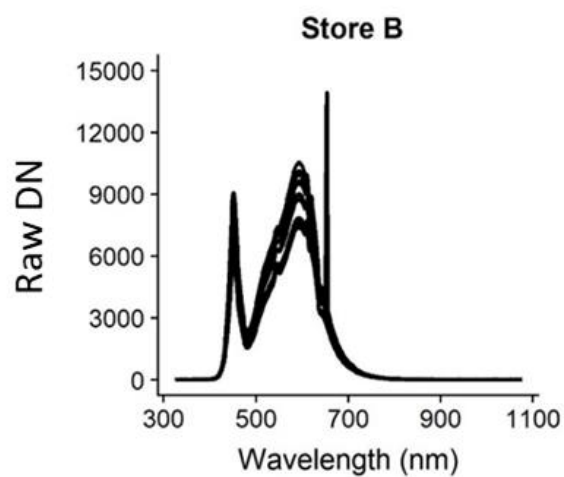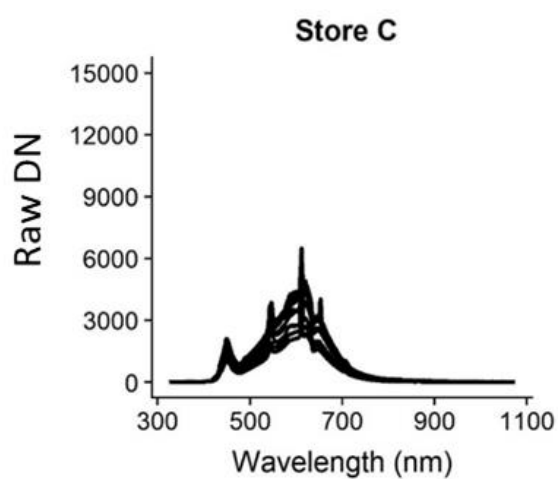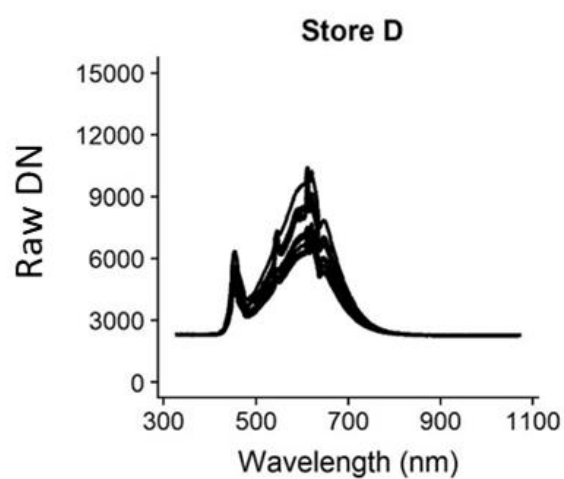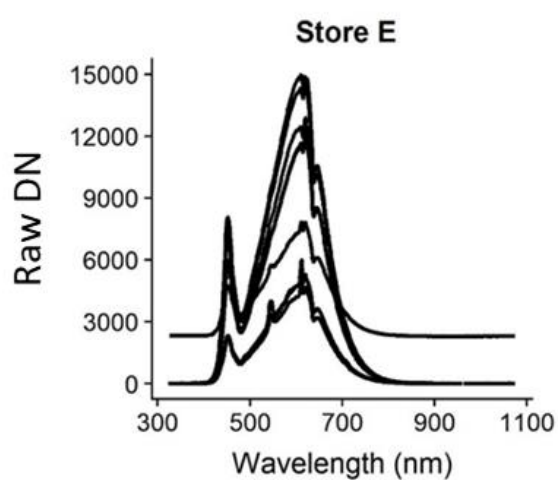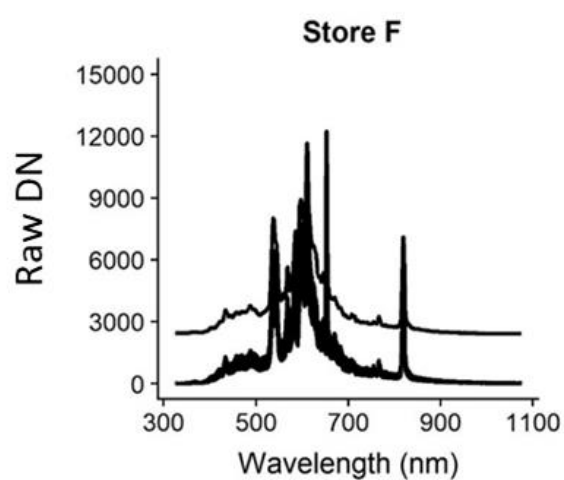

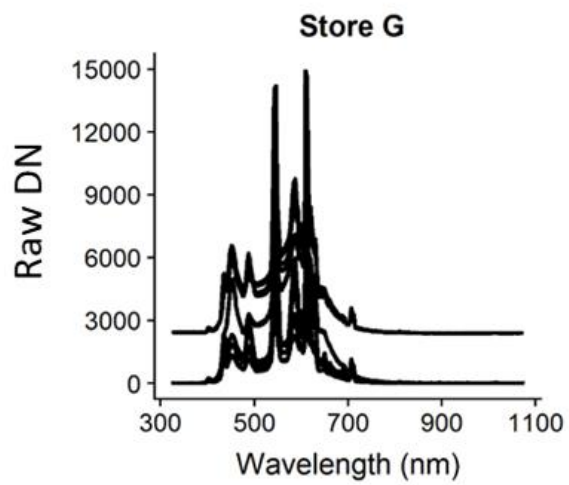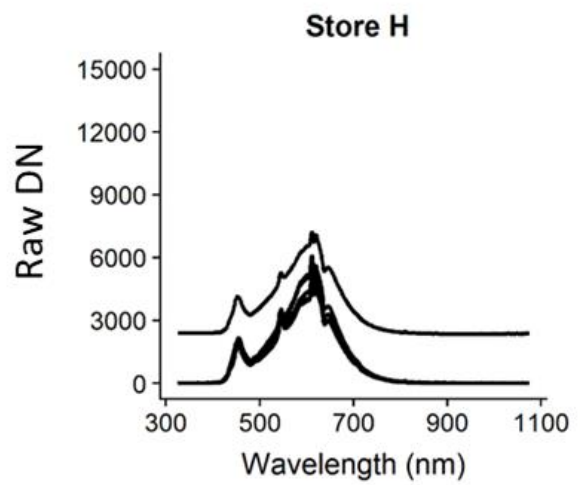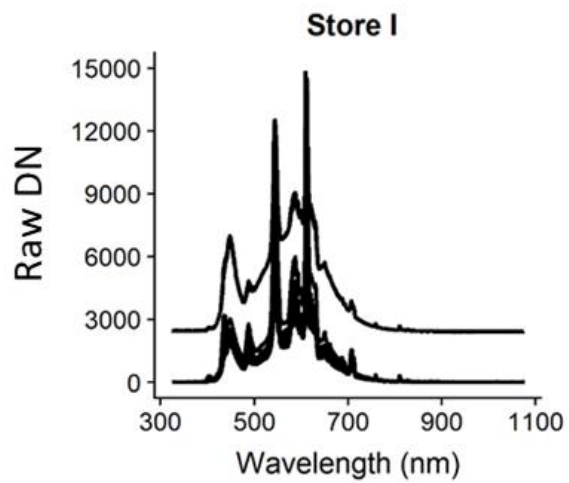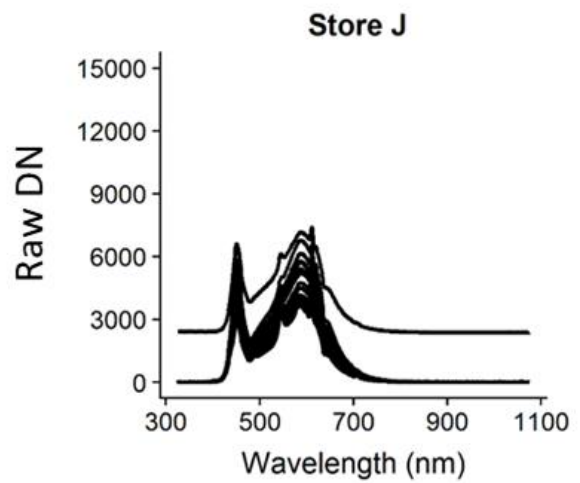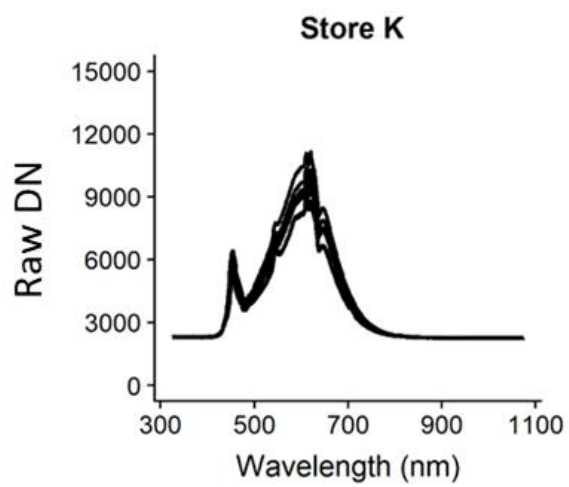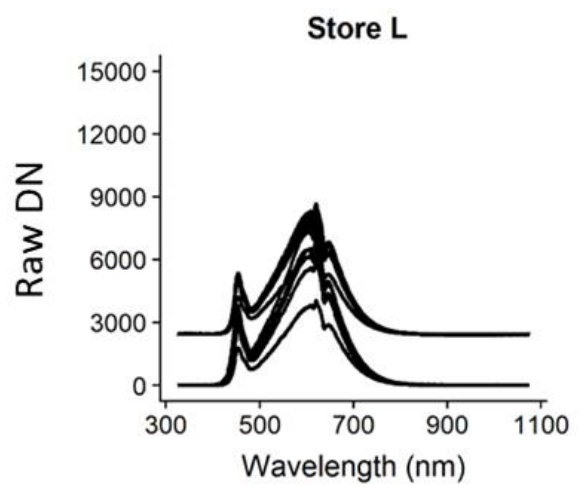

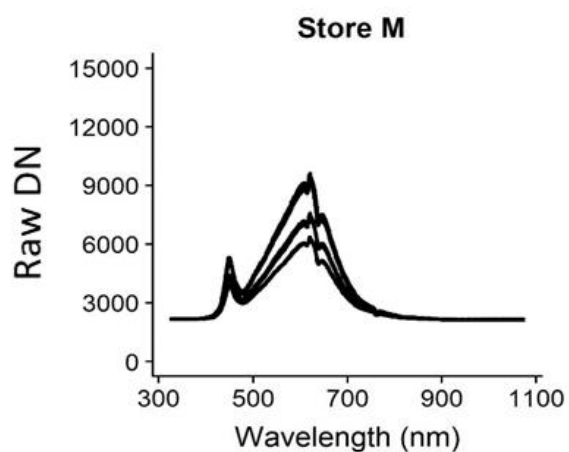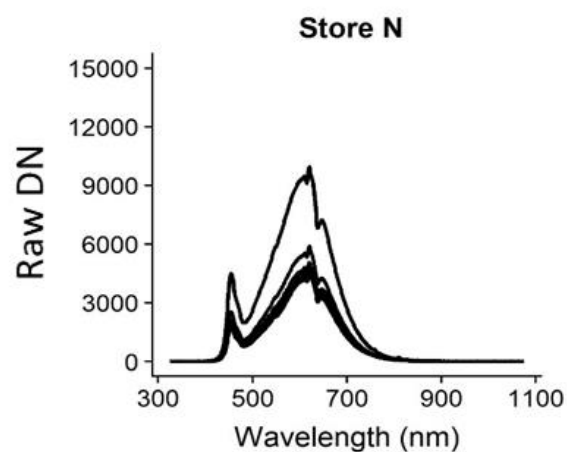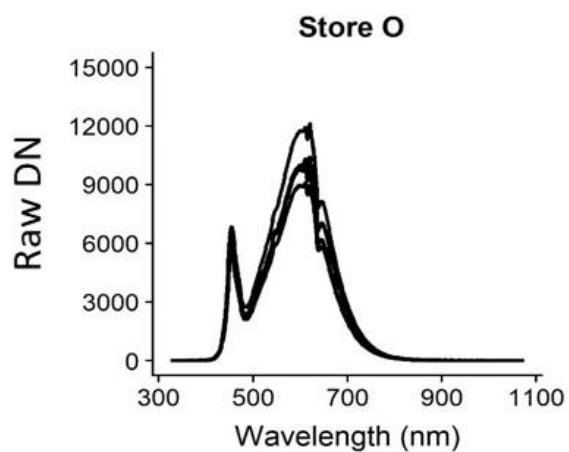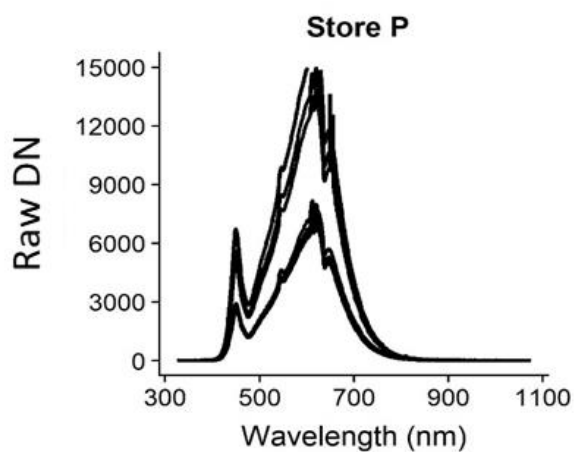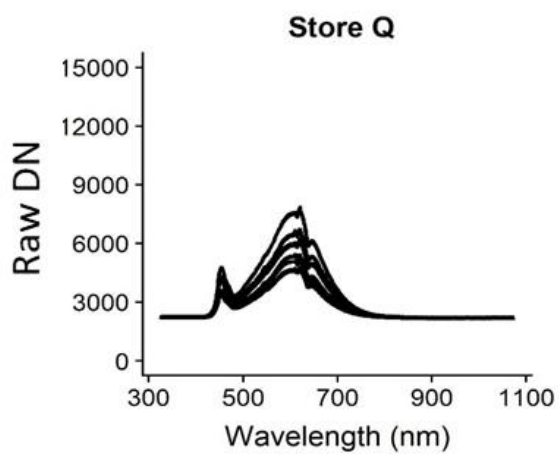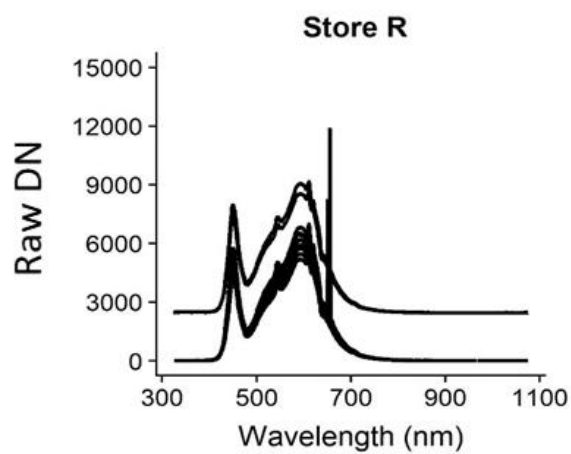

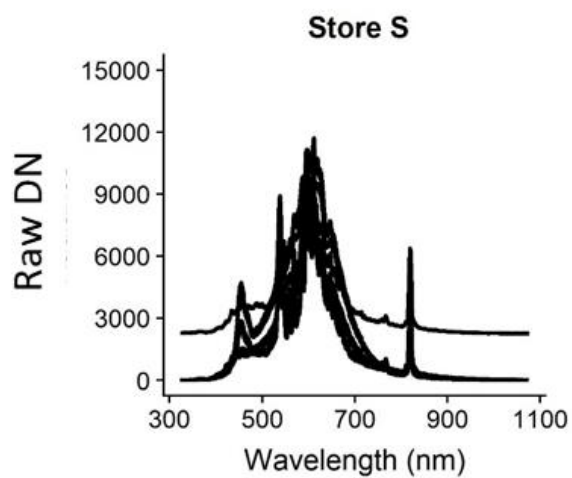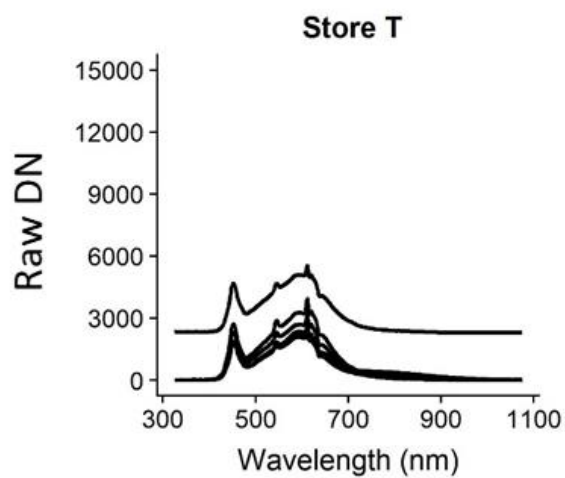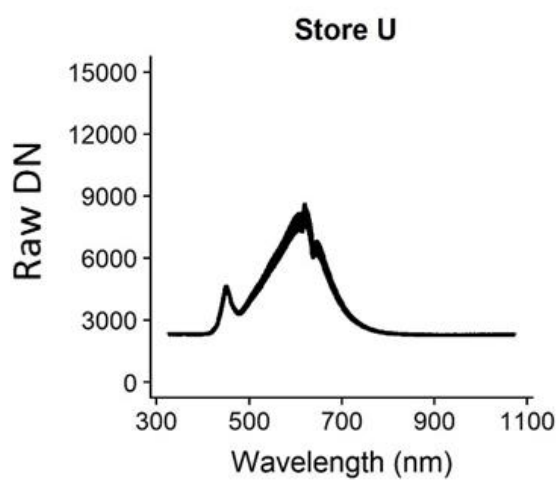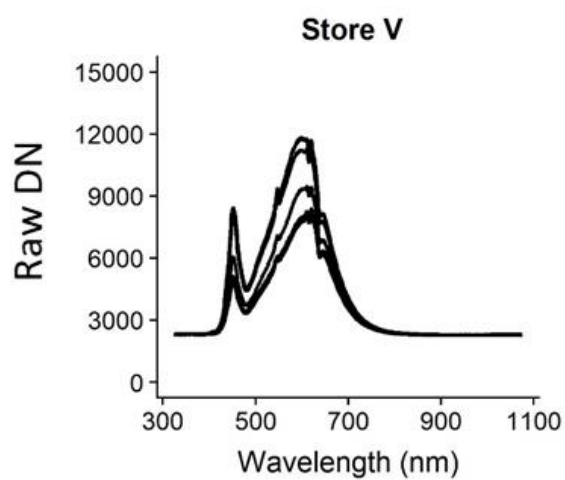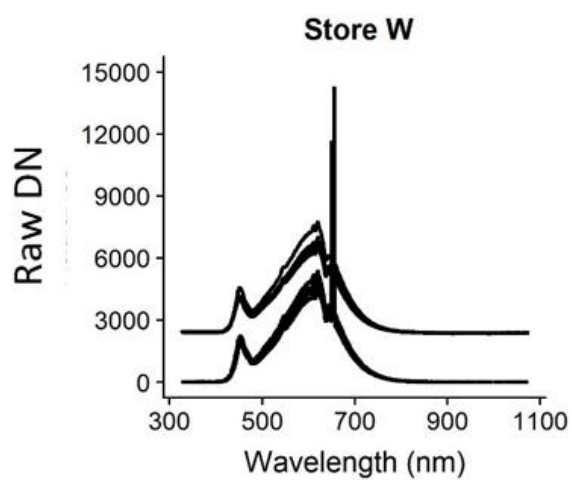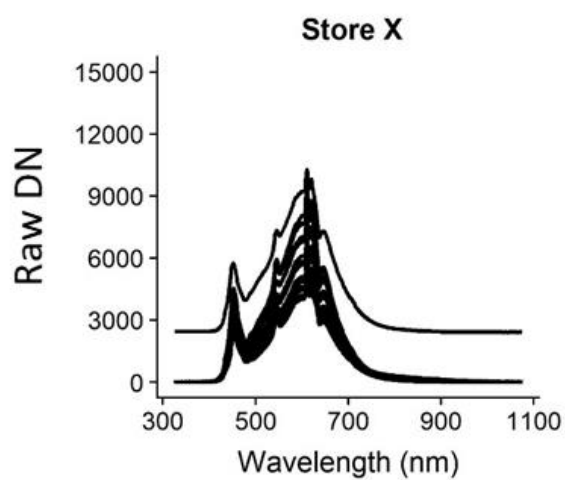

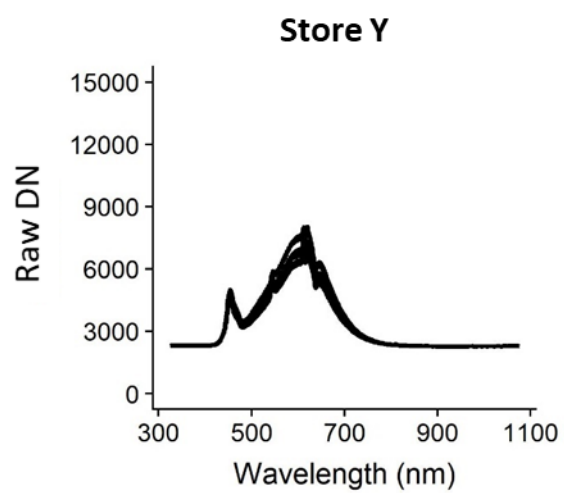

Supplement: S1 File — In each store, around 15 measurements were taken randomly around the potato display area, at shelf height, shown as multiple spectra in each graph. Raw DN refers to raw digital numbers. (PDF) [file pone.0235522.s005.pdf]
